# Autosomal recessive congenital cataract is associated with a novel 4-bp splicing deletion mutation in a novel C10orf71 human gene

**DOI:** 10.1186/s40246-023-00492-6

**Published:** 2023-05-13

**Authors:** M. Chograni, H. M. Alahdal, M. Rejili

**Affiliations:** 1 Faculté de Médecine de Tunis, Laboratoire Génétique Humaine, University Tunis El Manar, Tunis, Tunisia; 2 Department of Biology, College of Science, Princess Nourah Bint Abdulrahman University, Riyadh, Saudi Arabia; 3 Department of Life Sciences, College of Sciences, Al Imam Mohammad Ibn Saud Islamic University (IMSIU), Riyadh, 11623 Saudi Arabia

**Keywords:** Cataract, Splicing, C10orf71, Exome, Homozygosity mapping, Expression

## Abstract

Congenital cataract is one of the most genetically heterogeneous ocular conditions with different genes involved in its etiology. Here, we describe the analysis of a new candidate gene of a congenital bilateral cataract associated with polymalformative syndrome, moderate global developmental delay, microcephaly, axial hypotonia, intrauterine growth restriction and facial dysmorphism for two affected siblings. Molecular analysis included exome sequencing and genome wide homozygosity mapping revealed a region of homozygosity shared by the two affected siblings at 10q11.23. The new *C10orf71* gene was included in this interval and direct sequencing of this gene revealed an already described homozygous c. 2123T > G mutation (p. L708R) for the two affected subjects. Interestingly, we revealed in contrast a 4-bp deletion on the 3'-splicing acceptor site of intron 3-exon 4, namely defined as IVS3-5delGCAA. The *C10Orf71* gene expression analysis using RT-PCR showed an expression pattern in different fetal organs and tissues as well as in leukocytes and confirmed that the IVS3-5delGCAA deletion of the *C10orf71* gene is a splicing mutation responsible for the shortening of the C10orf71 protein in the two related patients. The *C10orf71* gene has not been described to date as associated to the autosomal recessive phenotype.

## Introduction

Cataracts are the most common cause of irreversible blindness and are defined as any opacity of the lens [1]. Around the world, 200,000 children are thought to be blinded by cataract, and each year, 20,000 to 40,000 newborns are born with developing bilateral cataract [2]. Inherited cataracts typically develop early in infancy and are sometimes mistaken for congenital conditions [3]. The underlying genetic properties of hereditary cataracts are exceedingly variable, and they have not yet been fully defined. Although X-linked inheritance and autosomal recessive inheritance are also possible, autosomal dominant inheritance is the most common pattern for cataracts of genetic origin [2]. Although they may be linked to other visual defects or be a part of multisystem genetic illnesses, inherited cataracts are most frequently standalone conditions [4, 5]. Congenital cataracts have been linked to abnormalities in more than 29 genes, 9 of which have an autosomal recessive inheritance pattern [6, 7]. Cataract formation in conjunction with more severe disorders may be caused by errors in genes governing fundamental metabolic pathways [8–11]. Regions of homozygosity (ROHs) are excellent proxy markers for the mutation itself in patients with recessive conditions because they frequently include the disease-causing mutation and come from a healthy heterozygous common ancestor [12]. This is especially true if the patient comes from a consanguineous pedigree. Additionally, homozygoty mapping (HM) can be used to identify situations of uniparental disomy and significant heterozygous deletions that result in hemizygous (and seemingly homozygous) genotypes [13, 14]. Due to these factors, HM has been used for many years in medical genetics as a technique to prioritize certain genomic regions for targeted mutational tests.

The objectives of this study were to use genetic tools such as exome sequencing and homozygoty mapping to analyze the candidate gene for a congenital bilateral cataract that is linked to polymalformative syndrome, moderate mental retardation, microcephaly, axial hypotonia, intrauterine growth retardation and facial dysmorphism. In a consanguine Tunisian family, we report a novel gene, *C10orf71*, with a novel splicing mutation that causes congenital cataract. Furthermore, a thorough description of the case's clinical characteristics and therapeutic approach is provided.

## Material and methods

### Patients

The family under study came from a small town in the province of Bizerte, North of Tunisia. Informed consent was obtained from participants after the protocol was previously approved by the Institutional Review Board of the Congenital and Hereditary Disorders Department at Charles Nicolle Hospital (Tunis, Tunisia), and we confirm that the whole study protocol was approved by the named IRB, and all methods were performed in accordance with the relevant guidelines and regulations. Blood samples (2–8 ml) from the probands and family members were then collected by venipuncture in heparin-coated vacutainers, and family history, pedigree, and clinical data were recorded.

**Fig. 1:**
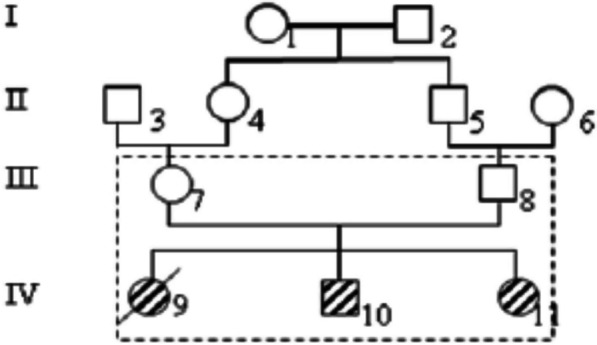
Genealogy of the congenital cataract family. Solid symbols designate affected subjects. Slash indicates deceased individuals. The squares and circles indicate male and female, respectively; numbers inside diamonds indicate the number of siblings from a specific individual or couple

A genealogical analysis revealed parental consanguinity (Fig. 1). According to reports, both parents had healthy vision well into their later years. In the first ten years of life, congenital cataracts were recorded in two siblings (Fig. 1), and all of them received ocular surgery at a young age (VI-10: at 6 months old and VI-11: at 1 year old). A control sample of 100 people was used, and a 9-week-old human embryo was used for RT-PCR.

### DNA extraction

Blood samples were collected from the subjects after obtaining their informed consent. Total DNA extraction from blood was performed using the phenol–chloroform. The integrity of 2% agarose gel electrophoresis showed that the main band of DNA was obvious, and there was no degradation. Using NanoDrop1000 spectrophotometer (Thermo Fisher, USA) to detect the purity and concentration, the purity of OD260/280 was 1.8–2.0, and the concentration was greater than 20 ng/ul.

### Exome sequencing

The exome was captured using the SureSelect Human All Exons v3 (20 patients) v4 (11 patients) and v5 (19 patients) reagents (Agilent Inc®). Sequencing was performed in an Illumina HiSeq 2000 instrument. Each exome library was indexed, separated into two equal halves and sequenced in two different lanes. Four half-libraries were sequenced in each HiSeq lane. The raw results were analyzed using a customized pipeline which utilizes published algorithms in a sequential manner (BWA for mapping the reads, SAMtools for detection of variants, Pindel for the detection of indels, ANNOVAR for the annotation). The entire coding sequence corresponding to the human RefSeq coding genes was used as the reference for the calculation of coverage and reads on target. All experiments were performed using the manufacturer’s recommended protocols without modifications.

### Genotyping to identify homozygous regions

DNA samples from affected family members, their unaffected siblings and their parents were received and genotyped using the HumanOmniExpress Bead Chip by Illumina Inc® (San Diego, CA, USA). This SNP array tests 720 K SNPs with a mean distance of 4 kb between the SNPs. After filtering for quality, the data were used in order to define the regions of homozygosity (ROH) for every individual using PLINK. We defined as homozygous regions, those regions with 50 consecutive homozygous SNPs irrespective of the total length of the genomic region, allowing for one mismatch. The ROH were further defined as genomic regions demarcated by the first encountered heterozygous SNPs flanking each established homozygous region. In order to delineate in each family, the target genomic region(s) potentially harboring the underlying pathogenic variant, we identified the ROH common to the affected individuals, excluding the ROH of the unaffected siblings and their parents.

### Bioinformatic analysis

Only homozygous exonic and splicing variants (± 6 bp of the intron–exon boundary) found within the target region of each family were retained for further analysis. Variants were initially filtered to exclude synonymous variants, variants with a minimum allele frequency 0.02 in dbSNP version 137, 1000Genomes, Exome Variant Server, our local database, variants found within segmental duplications of the genome and heterozygous variants. Variants remaining after the initial filtering process were evaluated based upon the predicted pathogenicity scores provided by SIFT, PolyPhen2 and Mutation Taster (2 out of 3 were required in order to declare a variant to be possibly pathogenic or possibly benign), their presence in the Professional version of HGMD and a literature search focusing on functional data. Evolutionary conservation scores were provided by PhyloP and GERP +  + . All filtering procedures and ROH processing were performed by a customized in-house algorithm.

The 3D structure of the mutated and the wildtype *C10orf71* proteins are determined using the Jmol plateform (https://www.rcsb.org/docs/3d-viewers/jmol), and the assed target RNA sequences are defined based on the SpliceAid server (http://www.introni.it/splicing.html).

### RT-PCR

In order to delimit the expression of *C10orf71* gene in different human cells, we assessed the mRNA *C10orf71* amplification in 13 foetal tissues of 9 weeks human embryo and leucocytes of healthy patient (control). To do so, an informed consent was obtained from a parent. The protocol was approved by the Institutional Review Board of the Congenital and Hereditary Disorders Department at Charles Nicolle Hospital (Tunis, Tunisia), and we confirm that the whole study protocol was approved by the named IRB, and all methods were performed in accordance with the relevant guidelines and regulations. Total cellular RNA from fresh human whole blood of foetal tissues and the control was carried out by means of the QIAamp RNA Blood Mini Kit *(Qiagen)*. An RT-PCR was performed using the QIAGEN® OneStep RT-PCR Kit and primers (F: 5’…ACACCGCCCCAGGAAACACC…3’ and R: 5’…GCAGGGCGAGGGAAGAGCAG…3’) selected from *C10orf71* mRNA.

## Results

### Clinical examination

Probands inclusion criteria were the presence of a congenital bilateral cataract associated with polymalformative syndrome (such as Microphthalmia, strabismus and nystagmus), moderate global developmental delay, microcephaly, axial hypotonia, intrauterine growth restriction and facial dysmorphism (Table 1). Regarding the patient’s development, the two sibs (IV_10_ and IV_11_) had delay in gross motor, fine motor, and cognition to a various extent (Table 1). The boy IV_10_ walked with assistance, sat with support for 5 months of age, and the two sibs started babbling only after 2 years of age. Their magnetic resonance image revealed the presence of Dandy-Walker anomaly associated with thinning of the corpus callosum. Karyotyping with R-banding revealed normal karyotypes: 46 XX for females, 46 XY for males. Bilateral cryptorchidism and an umbilical hernia were observed for the boy IV_10_. In the same siblings, we noted the death of the girl IV_9_ at the age of 3 years and who presented the same clinical signs as his brother IV_10_ and her sister IV_11_, in addition to hemorrhagic rectocolitis (UCH).

**Table 1:** Specialist examination of two patients

Patients	IV_10_	IV_11_
Weight (Kg)	3.8 DS (at 3 years)	3 DS (at 1 year)
Height (cm)	2DS (at 3 years)	9 DS (at 1 year)
Cataract	bilateral	bilateral
Age of cataract	6 months	1 year
Microcephaly	3.5 DS	3DS
Mental retardation	moderate	moderate
Psychomotor development	Spoke at 2 years Speech with monosyllabic words	
	Sit with support since 5 months Walk with assistance gained	–
Magnetic resonance image	Anomaly of Dandy-walker Thinning of corpus callosum	
Others	Polymalformatif syndrome: Microphthalmia, strabismus (isotropia), nystagmus Axial hypotonia Intrauterine growth retardation	
	Bilateral cryptorchidism operated Umbilical hernia	–

### Exome sequencing and homozygosity mapping

The exome sequencing carried out in the 2 patients and their parents generated a total of more than 22,000 genetic variants. The CGH array in the patients did not reveal any significant genomic deletion or duplication. Among all these variants, only the exonic and splicing-type variants (± 6 bp of the intron–exon junction), identified in the homozygous state in ROH (region of Homozygosity) shared between the 2 patients, were selected. The different selected variants were evaluated based on their pathogenicity prediction scores, their identification from the HGMD database and literature data, and their evolutionary conservation scores (homology prediction). We have therefore selected only one gene *C10orf71* (NM_199459: exon4: c.T2123G: p.L708R) with no score determined in the appropriate database. The selected gene has not been described to date as associated to the autosomal recessive phenotype.

### Sanger sequencing identified a novel mutation in C10orf71 gene from the homozygous region

**Fig. 2:**
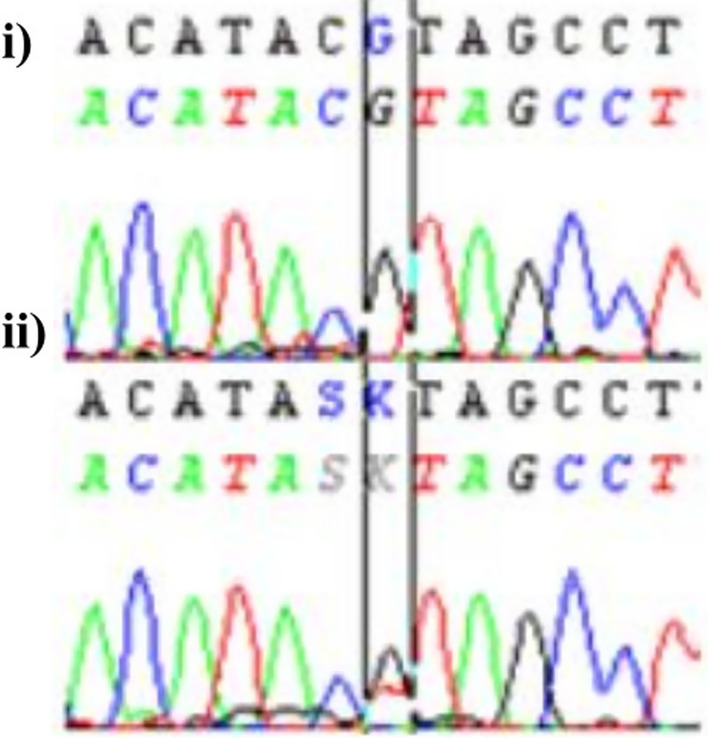
DNA sequencing chromatograms of the *C10orf71* missense variant c.2123 T > G identified in the 2 patients (i) and their parents (ii)

The sequencing analysis of the *C10orf71* coding exons and the exon–intron boundaries (splice site) identified a transversion of T by G at the level of exon 4 (Fig. 2) leading to leucine substitution for arginine at position 708 of the hypothetical protein C10orf71 (p. L708R).

**Fig. 3:**
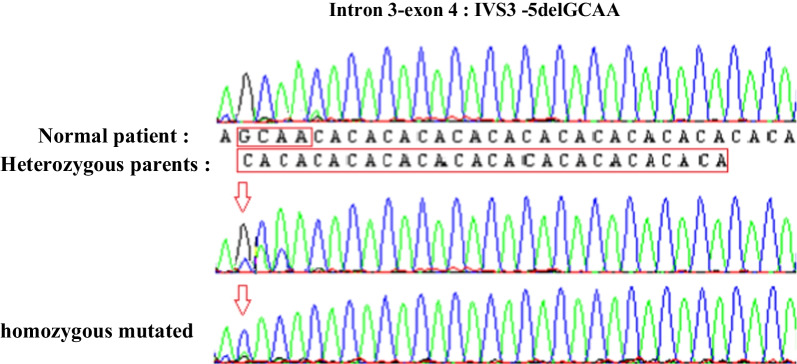
Mutational profile of the IVS3 -5delGCAA deletion located at the intron3-exon4 of the *C10orf71* gene in the two heterozygous normal parents (III_7_ and III_8_) and their two affected homozygous children (IV_10_ and IV_11_)

By focusing on the p. L708R, we realized that is not a new variation and it is a described SNP (rs20006285) in the 1000 Genomes project. Interestingly, we revealed in contrast a 4-bp deletion on the 3'-splicing acceptor site of intron 3-exon 4, namely defined as NM_199459:g.27781_27784delGCAA or IVS3 -5delGCAA, and identified as homozygous for the 2 patients (IV_10_ and IV_11_), heterozygous for the 2 parents (III_7_ and III_8_) and absent for 50 unrelated control individuals (Fig. 3).

### Prediction of the Novel 4-bp deletion effect

**Figure. 4:**
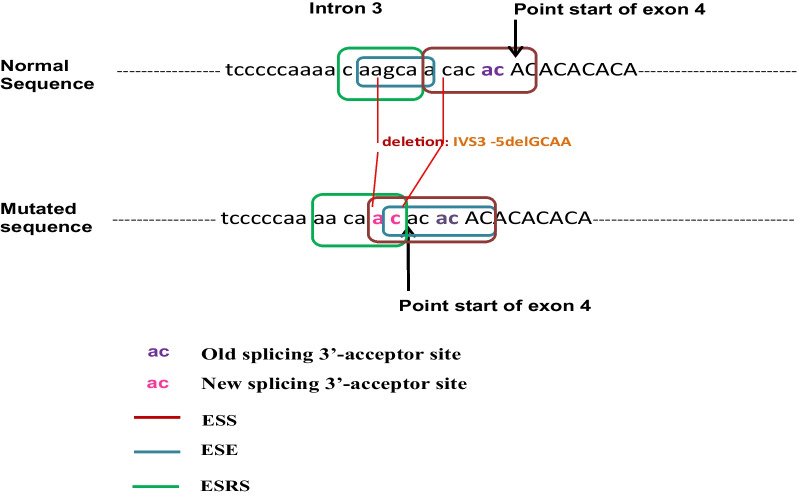
Schematic representation of the effect of the IVS3 -5delGCAA deletion of the C10orf71 gene on the splicing of exon4

**Fig. 5:**
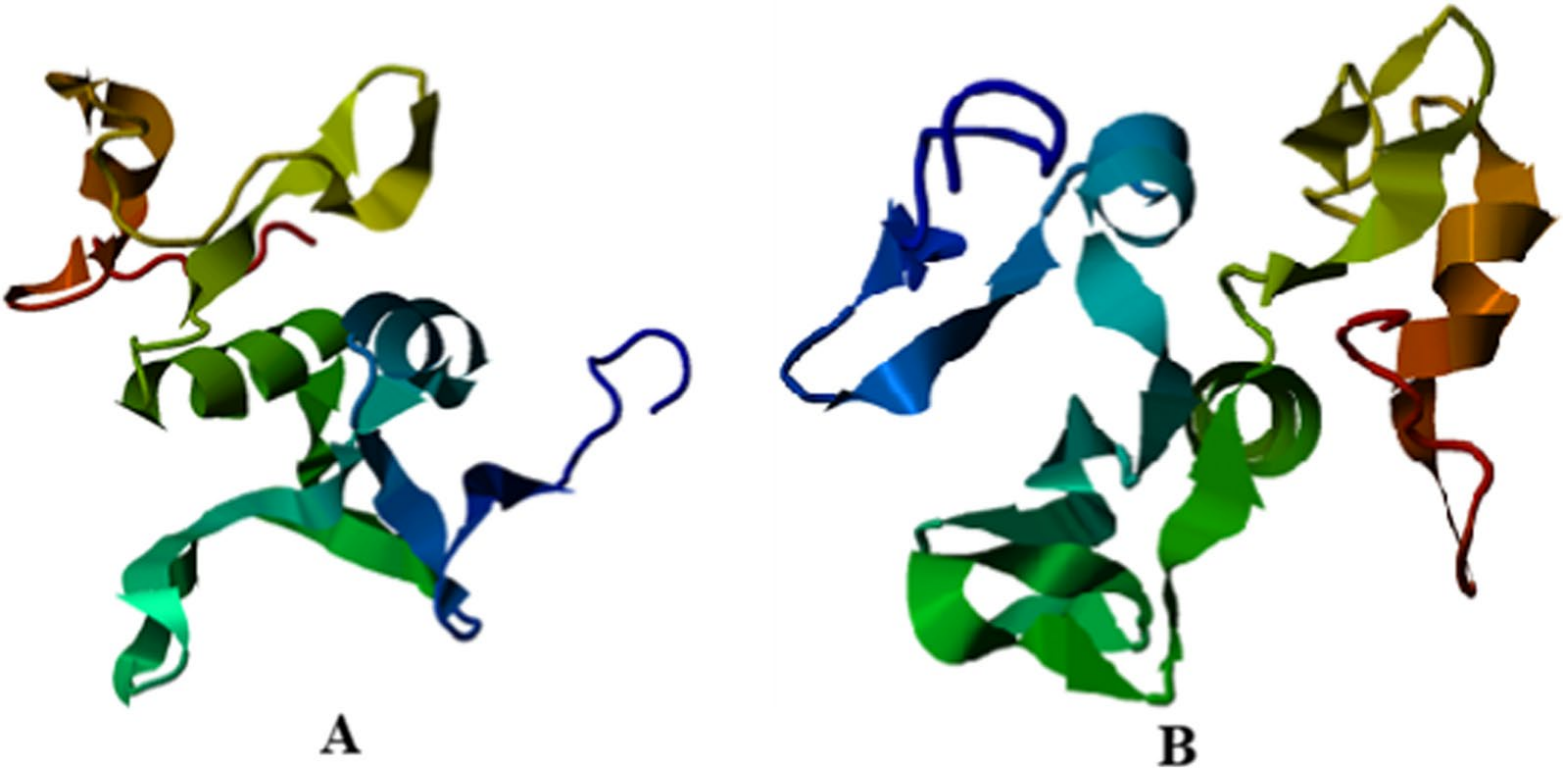
The predicted 3D structure of the normal (**A**) and mutated (**B**) the protein C10orf71

In order to predict the effect of IVS3-5delGCAA on the sequence of the *C10orf71* gene and in particular on the 3'-splicing acceptor site of intron 3-exon 4, the prediction results (HSF and ESE softwares) showed that GCAA deletion abolishes the AC acceptor splice site located at the 3' end of intron 3 and leads to the creation of a new cryptic site with 4 bp retention (ACAC) of intron 3 in the coding sequence of exon 4 (rich in AC) during pre-mRNA splicing (Fig. 4). Therefore, the generated cDNA would have a size of 2945 bp instead of 2941 bp (normal cDNA) due to the disruption of the 6 to 7 bases exonic splicing regulatory elements (ESRs) at the level of intron 3, known as inducers of the pre-mRNA splicing. At the protein level, IVS3-5delGCAA leads to a premature stop codon, resulting in a shortened protein (709 amino acids) that may function improperly, be nonfunctional, or get broken down. The predicted 3D structure of the normal and the mutated protein C10orf71 (p. L708R) is given in Fig. 5 (A and B).

### C10Orf71 gene expression analysis using RT-PCR

**Fig. 6:**
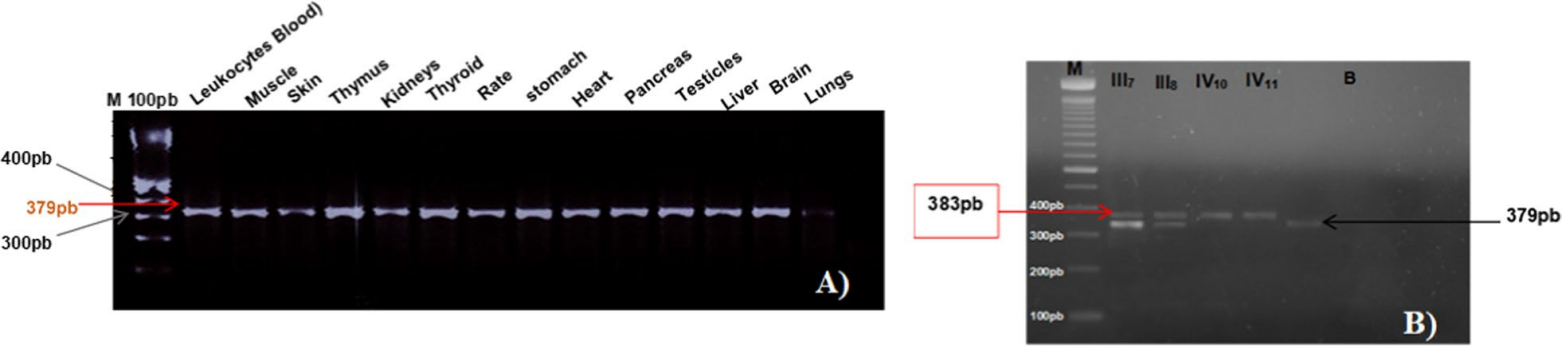
*C10orf71* gene PCR product. **A** the size of the amplicon (379 bp) in various fetal tissues, and leukocytes (blood) of unrelated control individual. **B** Polyacrylamide gel of the cDNA amplicon generated and amplified by RT-PCR showing the normal size of the amplicon for the positive control (379 bp) and for two affected patients IV_10_ and IV_11_ (383 bp)

Specific amplification of partial mRNA sequence encoding *C10orf71*gene from 13 fetal tissues extracted RNAs of a 9-week-old embryo, and leukocytes (blood) of unrelated control individual, revealed a partial band size of 379 bp for all different tested tissues (Fig. 6A*).* Consequently, the expression pattern of *C10orf71* gene in different fetal organs and tissues as well as in leukocytes is reported.

In order to highlight the difference of the target 4-bp deletion in the cDNA of the patients and their parents, we partially amplified the leukocyte cDNA region including the deletion. The observed PCR products on polyacrylamid gel showed two expected distinct sizes, 379 bp (unrelated individual, 1 band) and 383 bp for the 2 patients (IV_10_, IV_11_) homozygous for the deletion (1 band) (Fig. 6B), whereas the PCR product of the two parents (III_7_, III_8_), heterozygous for the deletion, generated two close bands corresponding to the 2 afro-mentioned sizes (Fig. 6B). Interestingly, our results confirm that the IVS3-5delGCAA deletion of the *C10orf71* gene is a splicing mutation responsible for the shortening of the C10orf71 protein in the 2 related patients.

### The pathological splicing mutation IVS3-5delGCAA causes a change in the target RNA sequences binding to the c10orf71 RNA sequence

**Fig. 7:**
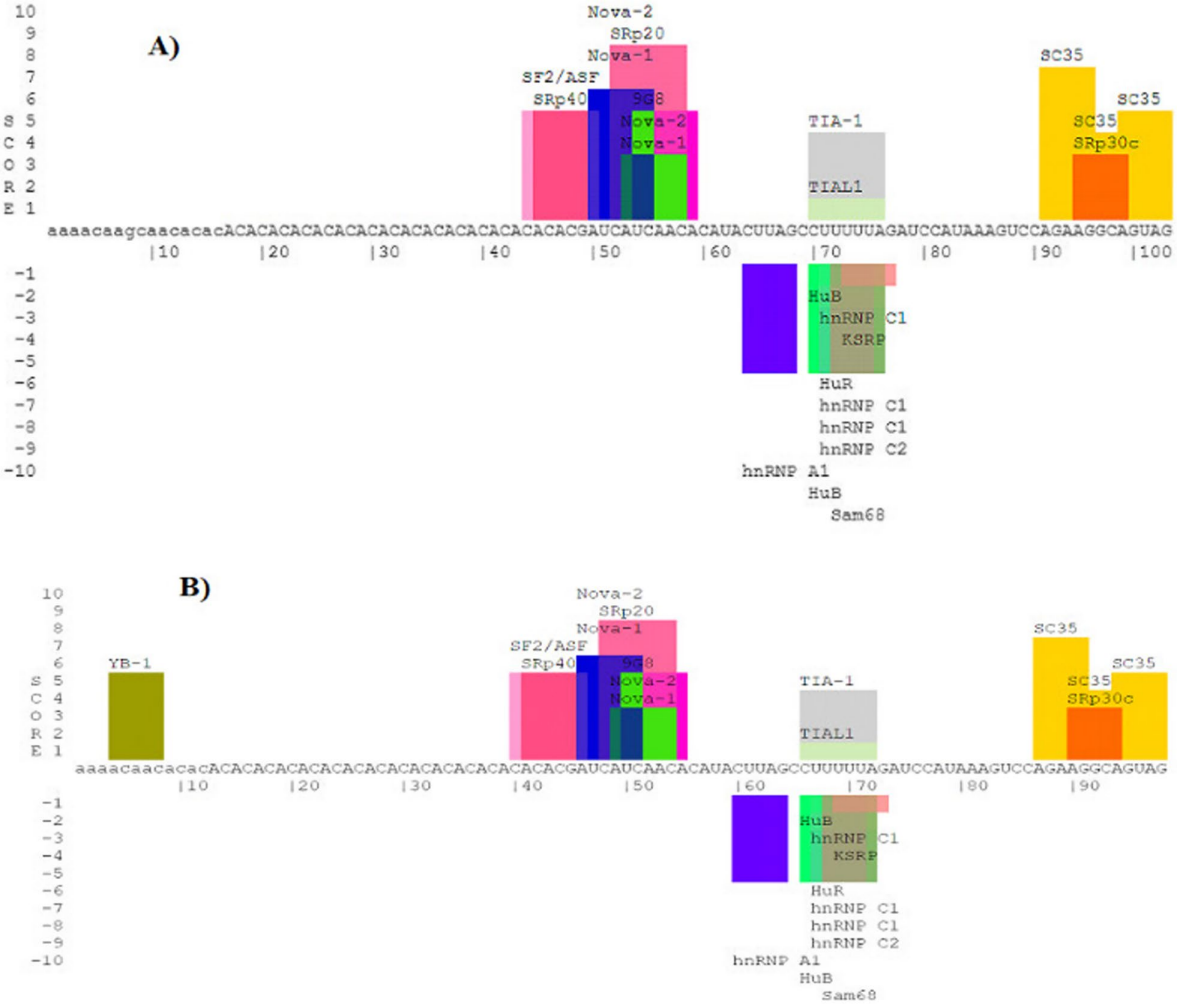
Assessed target RNA sequences that binds to the wild-type (**A**) and mutated (**B**) *c10orf71* RNA sequence in silico

Figure 7 displays the assessed target RNA sequences that bind the mutated and wild type *c10orf71* RNA sequence in silico. Interestingly, we report that A/C-rich region of the mutated *c10orf71* RNA sequence is recognized by the human YB-1 RNA sequence that was absent for the wild-type sequence. These observations suggest that the human YB-1 protein could participate in the recognition of exon enhancers and the maximal in vivo splicing mechanism for the affected patients.

## Discussion

Congenital cataracts are frequent, serious eye disorders that frequently result in neonatal blindness [15]. It could appear as a single aberration, a symptom of more widespread ocular development flaws, or a part of a multisystem illness [15]. As far as we know, this is the first report describing a more complex phenotype associated to the congenital cataract with a novel splicing deletion mutation in the new human gene *C10orf71*.

By clinical examination, the consanguineous analyzed family presented with 2 patients (the 3^rd^ is deceased) is characterized by a more complex phenotype. Similar to our results, many studies reported the association of cataract with congenital anomalies, global developmental delay and microcephaly in several cases [16–18]. In addition to congenital cataract, the 2 affected individuals had facial dysmorphism, which is not characteristic of a specific syndrome, growth retardation, anomaly of Dandy-Walker, axial hypotonia and polymalformative syndrome (microphthalmia, nystagmus, convergent strabismus). Dandy-Walker malformation (DWM) is the most common posterior fossa malformation, and it typically occurs sporadically [19]. Leyenaar et al. [20] reported that hypotonia may be the presenting sign for many systemic diseases and diseases of the nervous system. Interestingly, the association between these different signs constituted a very particular clinical picture not described in the literature, suggesting that it is probably a new syndrome associated with congenital cataract.

By exome sequencing and homozygoty mapping, our results reported a splicing variant in C10orf71 (10q11.23) gene localized on chromosome 10. At the time of writing, no study reported *C10orf71* as a candidate gene responsible for cataract phenotype. Based on different databases, *C10orf71* is an alias name of cardiac enriched FHL2 interacting protein and it is predicted to be involved in positive regulation of calcineurin-NFAT signaling cascade. Hojayev et al. [21] reported that FHL2 (four-and-a-half LIM domain protein 2) is expressed predominantly in the heart, and inactivation of the gene coding for FHL2 leads to exaggerated responsiveness to adrenergic stress. Cardiac-enriched FHL2-interacting protein (CEFIP) is a protein encoded by the gene *C10orf71* on chromosome 10 open reading frame 71. CEFIP is located at a structural component z-disk that is important for mechanical stability and contractility of both cardiac and skeletal muscles. Genomic studies of *C10orf71* revealed its specific expression in skeletal and cardiac muscle tissue. The novel cardiac z-disk protein CEFIP regulates cardiomyocyte hypertrophy by modulating calcineurin signaling [22]. Recently it was found that CEFIP overexpression is linked to favor overall survival in pediatric patients with Wilms tumor the most common type of kidney cancer in children identification of a five-mRNA signature as a novel potential prognostic biomarker in pediatric Wilms tumor [23]. However, no other links were associated with the gene nor the link between *C10orf71* and cataract has been addressed before. Interestingly, some clinic examinations reported that congenital cataract is linked to congenital heart disease (CHD) [24, 25]. Deloukas et al. [26] reported that 85 disease loci are human chromosome 10-borne. For CC and CHD, specific etiological causes, such as genetic alterations, have been identified. In monogenic variants of CC, more than 100 genes and over 200 loci have been found [27]. Additionally, some monogenic illnesses, copy number variation syndromes and illnesses connected to prenatal infections have cataract and heart problems concurrently present, such as oculo-facio-cardio-dental syndrome (OFCD) caused by BCOR mutations [28] and Down’s syndrome caused by trisomy 21 [29].

By direct sequencing, our results revealed that the two affected patients are homozygous for the detected variation, and their parents were heterozygous. Interestingly, the sequencing of exon 4 containing the variation revealed a deletion of 4 bp (GCAA) in intron 3. The IVS3-5delGCAA deletion is located 5 bases from the exon 4 starting site and the splicing variant is located ± 6 bp from the intron–exon junction. This deletion would, according to HSF and ESE, abolish the AC splice acceptor site located at the 3' end of intron3-exon4 and potentially affect RNA splicing by altering splicing regulatory elements (SREs) [30]. As a result, the cDNA of the 2 patients presented a size of 2945 bp with a truncated protein, compared to their healthy carrier parents with only 2941 bp. Interestingly, we report that A/C-rich region of the mutated *c10orf71* RNA sequence is recognized by the human YB-1 RNA sequence that was absent for the wild-type sequence. Many reports demonstrated that pathogenic nucleotide alterations can affect the detection of auxiliary cis-acting splicing regulatory regions by particular trans-acting proteins, leading to abnormal splicing. When present in exons, these regulatory elements are referred to as exonic splicing enhancers or silencers (ESE and ESS, respectively). Members of the SR-protein family facilitate spliceosome assembly at the splice sites typically recognize ESE. Other beneficial trans-acting elements, however, have also been discovered. For instance, it has been demonstrated that the YB-1 protein binds an A/C-rich region in the alternatively spliced v4 exon of the CD44 gene [31]. In Tunisia, Smaoui et al. [32] reported a HSF4 homozygous splice mutation in intron 12 (c.1327 + 4A > G) causing the skipping of exon 12 and leading to the ARCC phenotype in a consanguineous Tunisian family. In previous work, we reported the absence of mutations in four genes (*PAX6*, *PITX3,* *HSF4* and *LIM2* genes) encoding for congenital cataract and expressed in the human brain in Tunisian families with cataract and mental retardation [33].

In addition, our results reported a widely expressed of the *C10orf71* gene since it is detected in different fetal organs and tissues (muscle, skin, kidneys thymus, spleen, stomach, heart, pancreas, testicles, brain, thyroid, lungs and liver) as in leukocytes.

Our study provides further evidence for the importance of *C10orf71* gene in maintaining the normal structure and function of the lens and demonstrates for the first time that a mutation in *C10orf71* results in human ARCC. The fact that there are numerous difficult-to-model splicing systems [34] is clear that more understanding of the factors that influence splicing regulation is required. This would be very important for clinical diagnosis of human ARCC genetic diseases as well as for potential strategies to undo aberrant splicing events.

## Data Availability

The wild type and mutant *C10orf71* gene sequences datasets generated and/or analysed during the current study are available in the [GenBank] repository, [OQ575334-OQ575335]. The Genetic polymorphisms analysed during the current study are available in the [dbSNP] repository, [https://www.ncbi.nlm.nih.gov/SNP/snp_viewTable.cgi?handle=MREJ-SNP2023]. The *C10orf71* gene transcript was generated from the Ensembl under the reference C10orf71-201:ENST00000323868.
